# Propranolol treatment of infantile hemangioma endothelial cells: A molecular analysis

**DOI:** 10.3892/etm.2012.654

**Published:** 2012-08-03

**Authors:** JESSICA STILES, CLARISSA AMAYA, ROBERT PHAM, REBECCA K. ROWNTREE, MARY LACAZE, ARLYNN MULNE, JOYCE BISCHOFF, VICTOR KOKTA, LAURA E. BOUCHERON, DIANNE C. MITCHELL, BRAD A. BRYAN

**Affiliations:** 1 Department of Biomedical Sciences, Paul L. Foster School of Medicine, Texas Tech University Health Sciences Center, El Paso, TX;; 2 Klipsch School of Electrical and Computer Engineering, New Mexico State University, Las Cruces, NM;; 3 Department of Pediatrics, Paul L. Foster School of Medicine, Texas Tech University Health Sciences Center, El Paso, TX;; 4 Vascular Biology Program and Department of Surgery, Children’s Hospital Boston and Harvard Medical School, Boston, MA, USA;; 5 Department of Pathology, CHU Sainte-Justine, University of Montreal, Montreal, Quebec, Canada

**Keywords:** infantile hemangioma, propranolol, endothelial cells, angiogenesis

## Abstract

Infantile hemangiomas (IHs) are non-malignant, largely cutaneous vascular tumors affecting approximately 5–10% of children to varying degrees. During the first year of life, these tumors are strongly proliferative, reaching an average size ranging from 2 to 20 cm. These lesions subsequently stabilize, undergo a spontaneous slow involution and are fully regressed by 5 to 10 years of age. Systemic treatment of infants with the non-selective β-adrenergic receptor blocker, propranolol, has demonstrated remarkable efficacy in reducing the size and appearance of IHs. However, the mechanism by which this occurs is largely unknown. In this study, we sought to understand the molecular mechanisms underlying the effectiveness of β blocker treatment in IHs. Our data reveal that propranolol treatment of IH endothelial cells, as well as a panel of normal primary endothelial cells, blocks endothelial cell proliferation, migration, and formation of the actin cytoskeleton coincident with alterations in vascular endothelial growth factor receptor-2 (VEGFR-2), p38 and cofilin signaling. Moreover, propranolol induces major alterations in the protein levels of key cyclins and cyclin-dependent kinase inhibitors, and modulates global gene expression patterns with a particular affect on genes involved in lipid/sterol metabolism, cell cycle regulation, angiogenesis and ubiquitination. Interestingly, the effects of propranolol were endothelial cell-type independent, affecting the properties of IH endothelial cells at similar levels to that observed in neonatal dermal microvascular and coronary artery endothelial cells. This data suggests that while propranolol markedly inhibits hemangioma and normal endothelial cell function, its lack of endothelial cell specificity hints that the efficacy of this drug in the treatment of IHs may be more complex than simply blockage of endothelial function as previously believed.

## Introduction

Infantile hemangiomas (IHs) are the most common benign tumors in infancy affecting 5–10% of the population, and are largely composed of densely packed over-proliferating capillaries with high cellular density and the absence of an open lumen. These lesions are most prevalent in Caucasian children and are three times more common in female infants than male. The head and neck region is the most frequently involved area (60%), followed by the trunk (25%) and the extremities (15%), and these tumors exhibit a non-random distribution largely correlating with regions of embryological fusion (1). IHs have a predictable natural history, arising soon after birth to undergo a significant proliferative phase during the first year of life, followed by gradual involution over several years. Regression is complete in 50% of 5-year-old patients and 90% of 9-year-old patients. Because these tumors spontaneously regress and (for the majority of lesions) produce no long-term scarring, most children with IHs require no treatment. Despite their self-limiting course, in approximately 10% of cases depending on their anatomical site and/or size, there can be serious or life threatening complications requiring immediate intervention. The classical approaches for treating complicated IHs include the use of systemic or intralesional corticosteroids, chemotherapeutic agents such as vincristine, laser therapy, surgical resection or a combination of these treatments. The recent serendipitous discovery of β blockers as an effective therapy for IHs has revolutionized management of IHs to become the current gold standard (2).

Propranolol, which is administered systemically in pediatric patients with IHs, is a non-selective β-adrenergic receptor antagonist that blocks the action of epinephrine and norepinephrin. This drug has been shown to suppress angiogenesis via inhibition of proliferation, migration, barrier function, and induction of apoptosis in primary cultures of normal epithelial cells (3–6). The molecular mechanism of action for propranolol includes disrupting the epinephrine and norpinephrine regulation of cyclic AMP production, actin cytoskeletal dynamics and release of atherogenesis regulators (7–9). Only recently have investigations into the precise roles of propranolol in IHs revealed that this therapy blocks hemangioma endothelial growth and this effect may be through suppressing the production of nitric oxide and HIF1α regulation of vascular endothelial growth factor (VEGF) expression (10,11). Perplexingly, it is unknown how propranolol preferentially inhibits the growth of IHs, while spares the formation of new blood vessels necessary for growth and development of the infant. In this study, we sought to further evaluate the molecular mechanisms by which propranolol exerts its effects on human IH endothelial cells (HemECs). Furthermore, we compared the biological response of HemECs treated with propranolol to that of normal human endothelial cells treated with propranolol. Our data indicate that propranolol disrupts cell proliferation through modulation of key cell cycle regulators and blocks cell migration via alterations in the activation status of proteins essential for cytoskeletal dynamics. We further showed via microarray analysis that propranolol leads to large-scale changes in global gene expression, particularly in genes involved in lipid/sterol metabolism, cell cycle regulation, angiogenesis and post-translational modification. Interestingly, our data indicate that the effects of propranolol on HemECs are similar to that observed in normal endothelial cells, suggesting that this drug is not specific to HemECs.

## Materials and methods

### Cell lines and culture conditions

HemECs were previously isolated from proliferating-phase IH specimens collected from female infants (12). Primary cultures of neonatal human dermal microvascular endothelial cells (HDMVECs) and human coronary artery endothelial cells (HCAECs) were purchased from ATCC. These cell lines were cultured in vascular cell basal media (ATCC #PCS-100-030) and supplemented with 0.2% bovine brain extract, 5 ng/ml human epidermal growth factor, 10 mM L-glutamine, 0.75 U/ml heparin sulfate, 1 μg/ml hydrocortisone, 50 μg/ml ascorbic acid, 2% fetal bovine serum and penicillin/streptomycin. For all experiments cell lines were used at <10 passages.

### RT-PCR

RNA was isolated from cells using the Ambion Purelink Mini kit according to the manufacturer’s directions. qRT-PCR was performed on an ABI7900HT RT-PCR system using TaqMan assays with predesigned primer sets for the genes of interest (Invitrogen). All RT-PCR experiments were performed in triplicate. Data shown are the average RQ value ± standard deviation of 4 replicates.

### Western blot analysis

Cell lysates were collected after 48 h treatment and subjected to SDS-PAGE on gradient (4–15%) gels and subsequently transferred to PVDF for western blotting. p-vascular endothelial growth factor receptor-2 (VEGFR-2) (Cell Signaling #2478), VEGFR-2 (Cell Signaling #2479), p-p38 (Cell Signaling #4511), p-p44/42 (Cell Signaling #4370), p-SAPK/JNK (Cell Signaling #4668), p-ATF2 (Cell Signaling #5112), actin (Santa Cruz #SC47778), cyclin A1 (Abcam #ab13337), cyclin A2 (Abcam #7956), cyclin B2 (Abcam #18250), cyclin D1 (Cell Signaling #2978), cyclin D2 (Cell Signaling #3741), cyclin D3 (Cell Signaling #2936), cyclin E1 (Cell Signaling #4129), p15 (Cell Signaling #4822), p21 (Cell Signaling #2947), p27 (Cell Signaling #3698), cleaved caspase-9 (Cell Signaling #9509), cleaved caspase-3 (Cell Signaling #9664), p-FAK (Cell Signaling #3283), p-cofilin (Cell Signaling #3313), cofilin (Cell Signaling #3318), p-ERM (Cell Signaling #3149), ERM (Cell Signaling #3142), p-MYPT1 (Cell Signaling #4563) and MYPT1 (Cell Signaling #2634) antibodies were used at a 1:1000 dilution, followed by incubation with 1:1000 HRP-conjugated anti-mouse or anti-rabbit antibodies (as appropriate). Proteins were detected using Supersignal West Dura Extended Duration Substrate (Thermo Scientific) and digitally captured using a GE Image Quant LAS4000 imaging system.

### Proliferation assay

Cells were plated at subconfluent density and subjected to the indicated treatments for 48 h. Images from 5 independent areas were collected at 1-h intervals using a Nikon Biostation CT time lapse imaging robot. Changes in cell density were calculated every 24 h by counting the number of cells in the selected field of vision. Data shown represent the average of 5 independent areas ± the standard deviation.

### Flow cytometry

Cells were treated as indicated, trypsinized, and fixed in 70:30 ethanol:phosphate-buffered saline overnight. Cells were then stained with 200 μg/ml ethidium bromide plus 20 μg/ml RNase A and incubated overnight. DNA content was analyzed using an Accuri C6 flow cytometer. Data shown are representative of at least 3 independent experiments. Quantitative analysis of DNA content was performed using CFlow Plus software (Accuri) and is the average of triplicate data points.

### Live/dead assay

Cells were treated as indicated, stained for 10 min with 5 μg/ml Hoechst and 5 μg/ml propidium iodide, and washed 3 times in PBS. A Nikon C2SI scanning laser confocal microscope was used to image the red and blue channels. Percent apoptosis (A) was calculated by the following formula: A = (number of red cells/number of blue cells) x 100. The data presented is the average of triplicates.

### Migration assay

Confluent cultures were treated as indicated, scratch wounded, and the progress of ‘healing’ of the wound was monitored using a Nikon Biostation CT time lapse imaging robot. Migration speed was calculated by monitoring the movement of the ‘wound’ toward its center at each hour over a 12-h period.

### Immunofluorescence and cytoskeletal organization calculations

Cells were grown on glass coverslips, treated as indicated and fixed in 4% paraformaldehyde. Then, the coverslips were blocked in 5% bovine serum albumin plus 0.5% Tween-20, incubated with 1:200 of the p-FAK antibody and detected with fluorescently conjugated secondary antibodies. Actin microfilaments were detected by staining with Rhodamine-conjugated phalloidin, and cell nuclei were detected with 4′,6-diamidino-2-phenylindole (DAPI). Immunofluorescent images were captured as z-stacks using a Nikon C2SI scanning laser confocal microscope. Image analysis of cytoskeletal organization included calculating the actin stress fiber correlation and binarizing this correlation image to determine fiber lengths using the FiberScore algorithm (13).

### Microarray analysis

Total RNA was amplified and biotin-labeled using Illumina TotalPrep RNA Amplification kit (Ambion). A total amount of 750 ng of biotinylated aRNA was then briefly heat-denatured and loaded onto expression arrays to hybridize overnight. Following hybridization, arrays were labeled with Cy3-streptavidin and imaged on the Illumina ISCAN. Intensity values were transferred to Agilent GeneSpring GX microarray analysis software and data were filtered based on the quality of each call. Statistical relevance was determined using ANOVA with a Benjamini Hochberg FDR multiple testing correction (p<0.05). Data were then limited by fold change analysis to statistically relevant data points demonstrating a 2-fold or more change in expression.

## Results

### β-adrenergic receptor expression in HemECs and IHs

**Figure 1:**
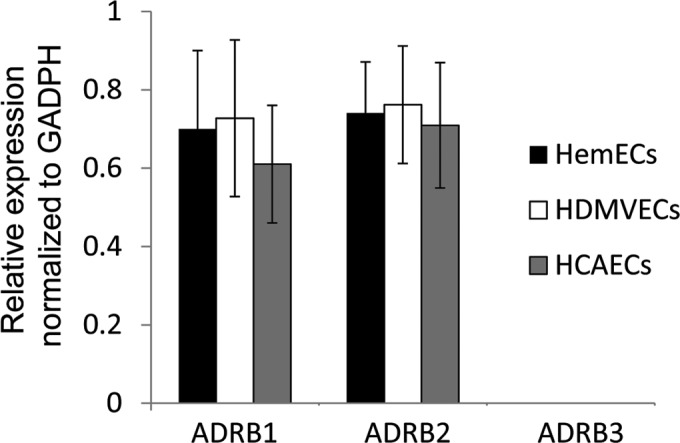
β-adrenergic receptor expression on infantile hemangioma (IH) and normal endothelial cells. RT-PCR expression assays measuring the steady state levels of ADRB1, ADRB2, and ADRB3 mRNA in primary cultures of human infantile hemangioma endothelial cells (HemECs), human dermal microvascular endothelial cells (HDMVECs) and human coronary artery endothelial cells (HCAECs). Expression data are represented as the relative abundance of each transcript normalized to the GAPDH levels.

The presence of β-adrenergic receptors on normal human endothelial cells has been previously confirmed (14). However, despite the extensive use of systemic propranolol as an anti-IH agent, the expression of the three known β-adrenergic receptors in endothelial cells isolated from these benign tumors is unknown. Using RT-PCR, we evaluated the steady state mRNA expression of the three known β-adrenergic receptors (ADRB1, ADRB2 and ADRB3) in cultured HemECs. Our data revealed that ADRB1 and 2, although not ADRB3, were expressed in HemECs (Fig. 1). Moreover, similar results were observed for both HDMVECs and HCAECs, with equivalent levels of each receptor across the three endothelial cell lines.

### Propranolol disrupts cell cycle progression

**Figure 2:**
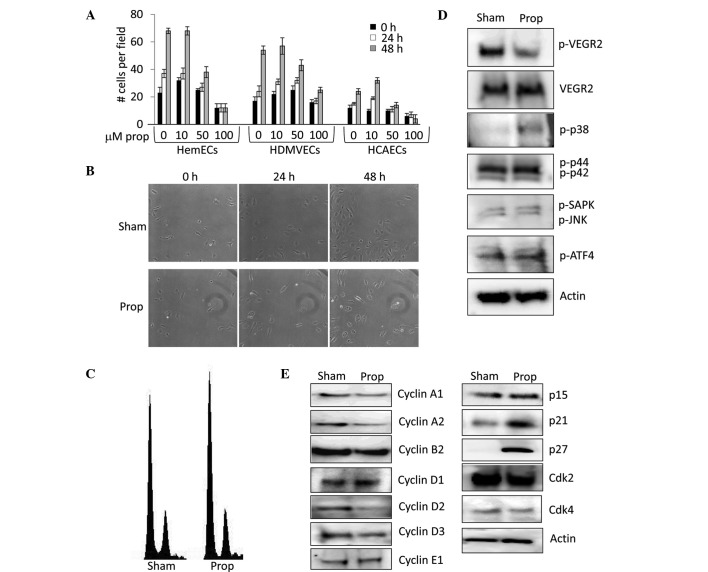
Propranolol decreases the proliferation of human infantile hemangioma endothelial cells (HemECs). (A) HemECs, human dermal microvascular endothelial cells (HDMVECs), and human coronary artery endothelial cells (HCAECs) were treated with a dose curve of propranolol (0 to 100 μM) and cell proliferation was measured by counting changes in the number of cells/defined vision field over a 48-h period. (B) Time lapse microscopy image of sham and 50 μM propranolol treated HemECs over a 48-h period. (C) DNA content analysis of propidium iodide stained HemECs treated with sham or 50 μM propranolol for 48 h. (D) Western blot analysis detecting the levels of phosphorylated and total vascular endothelial growth factor receptor-2 (p-VEGFR-2 and VEGFR-2, respectively) and the phosphorylated forms of p38 (p-p38), p44 (p-p44), p42 (p-p42), stress activated protein kinase (p-SAPK), c-jun N-terminal kinase (p-JNK), and activating transcription factor 4 (p-ATF4) in HemECs treated for 24 h with sham or 50 μM propranolol. Actin levels were used as a loading control. (E) Western blot analysis detecting the levels of cyclins, cyclin dependent kinases, and cyclin dependent kinase inhibitors in HemECs treated for 24 h with sham or 50 μM propranolol. Actin levels were used as a loading control. Prop, propranolol.

Despite the extensive use of propranolol, many of the mechanisms of action of this drug on IHs have been inferred from its effects on normal endothelial cells (7–9). Recent studies have suggested that propranolol may inhibit IHs by suppressing production of nitric oxide and HIF1α signaling (10,11). However, the signaling intermediates and many of the downstream effectors which modulate its action on IHs are largely unknown. To elucidate the molecular components at play following propranolol-induced inhibition of HemEC proliferation, we first examined the growth rates of HemECs, HDMVECs and HCAECs after treatment with a dose curve of propranolol. Our data indicate that the IC_50_ for propranolol-induced inhibition of proliferation was ∼50 μM for all three cell types (Fig. 2A and B), therefore for all subsequent experiments in this study we continued to use this concentration. Moreover, cell cycle analysis on the panel of endothelial cells using flow cytometry revealed that propranolol indiscriminately induced an increase in the proportion of cells in the G1 phase of the cell cycle, while reducing the proportion of cells in the S and G2/M phases (Fig. 2C, Table I). Equivalent results were observed in HDMVECs and HCAECs (Table I). To address the effects of propranolol on HIHEC proliferation, we analyzed the expression/activation status of a number of proteins involved in regulating proliferation. VEGFR-2 is a strong mitogenic regulator of endothelial cells that shows aberrant constitutive activation in HemECs (15), and its phosphorylation is reportedly blocked following propranolol treatment (5). Indeed, the 24-h treatment of HemECs with 50 mM propranolol resulted in sharply decreased VEGFR-2 phosphorylation (Fig. 2D). The mitogen activated protein kinases (MAPKs) are direct downstream effectors of VEGFR-2 regulating VEGF-induced endothelial proliferation. We tested the activation status of p38, p44, p42, SAPK, JNK, and the downstream effector ATF4 in HemECs experiencing 24 h of propranolol treatment. Of the proteins tested, the stress activated p38 (but not the stress activated SAPK or JNK) was the only one that exhibited a significant change in phosphorylation following propranolol treatment of HemECs (Fig. 2D). These data suggest that despite the inhibition of VEGFR-2 noted following the treatment, the major proliferative MAPKs such as p44 and p42 were not affected by propranolol treatment in HemECs. As flow cytometry analysis indicated that propranolol induced alterations in cell cycle progression, we performed western blot analysis on a panel of cell cycle regulators, discovering that this drug decreases the expression of key cyclin proteins (cyclins A1, A2, B2, D2 and D3) and increases the expression of important cell cycle inhibitors (p15, p21 and p27) (Fig. 2E). No change in the expression of Cdk2 or Cdk4 was observed following the treatment. These alterations in key cell cycle regulators likely account for propranolol-induced alterations in HemEC proliferation.

### It is speculated that propranolol may increase apoptosis of IHs

**Figure 3:**
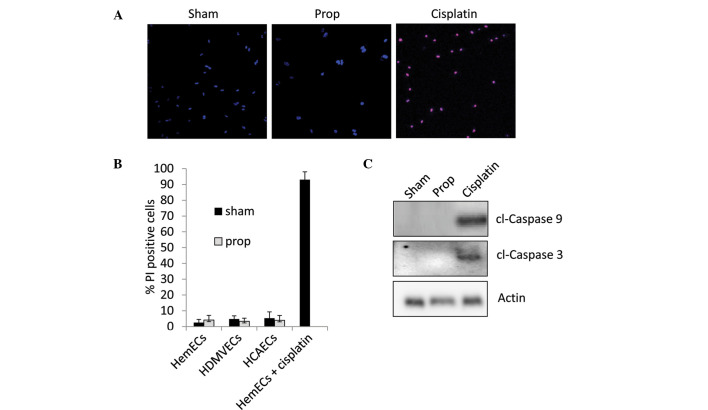
Propranolol does not induce apoptosis in human infantile hemangioma endothelial cells (HemECs) at its effective inhibitory concentration. (A) Confocal imaging of HemECs treated for 72 h with sham or 50 μM propranolol and subsequently co-stained with propidium iodide (PI) and Hoechst dye (blue, Hoechst-positive nuclei; pink, Hoechst-positive/PI-positive nuclei). (B) Quantification of PI-positive nuclei in HemECs, human dermal microvascular endothelial cells (HDMVECs), and human coronary artery endothelial cells (HCAECs) treated for 72 h with sham or 50 μM propranolol. (C) Western blot analysis detecting the levels of cleaved caspase-9 and -3 (cl-caspase-9 and cl-caspase -3, respectively). Actin levels were used as a loading control.

To determine whether this drug affects apoptosis, we treated HemECs, HDMVECs and HCAECs with 50 μM propranolol for 3 days. As a control we treated HemECs with 5 μM cisplatin for an equivalent amount of time. Cells were co-stained with propidium iodide (which only stains the nuclei of dead cells) and Hoechst dye (which stains the nuclei of both live and dead cells). Calculation of the apoptotic index from each treatment revealed that 50 μM propranolol did not induce apoptosis of any of the cell lines tested, while 5 μM cisplatin resulted in almost 100% apoptosis (Fig. 3A and B). We did observe significant apoptosis in all of our cell lines at concentrations of propranolol higher than 150 μM (data not shown). To confirm our observations, we utilized western blotting to detect the cleavage products of the apoptotic initiator caspase-9 and the apoptotic effector caspase-3. Our data indicate that while 5 μM cisplatin strongly induced caspase cleavage, 50 μM propranolol failed to induce apoptotic signaling (Fig. 3C).

### Propranolol disrupts cell migration and actin cytoskeleton dynamics

**Figure 4:**
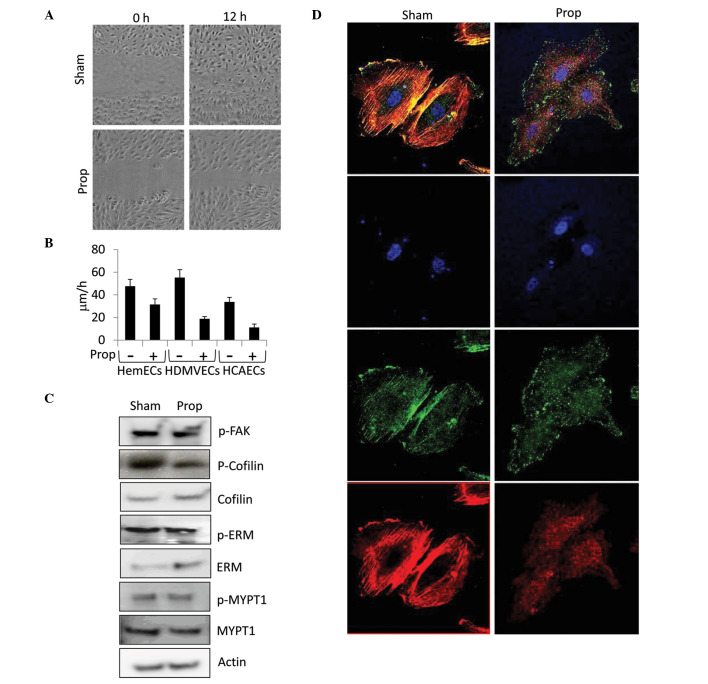
Propranolol disrupts HIHEC migration and actin cytoskeleton dynamics. (A) Confluent monolayers of human infantile hemangioma endothelial cells (HemECs) were scratch wounded and treated with sham or 50 μM propranolol. Progress of migration was monitored using time lapse photography over a period of 12 h. (B) Quantification of the speed (μm/h) of HemECs, human dermal microvascular endothelial cells (HDMVECs), and human coronary artery endothelial cells (HCAECs) treated with sham or propranolol from the time lapse images of the scratch assay. (C) Western blot analysis detecting the levels of the total and phophorylated (p-) forms of focal adhesion kinase (FAK), cofilin, ezrin/radixin/moesin (ERM), and myosin phosphatase-targeting subunit 1 (MYPT1) in HemECs treated with sham or 50 μM propranolol for 48 h. Actin levels were used as a loading control. (D) Confocal immunofluorescent imaging of sham or 50 μM propranolol-treated HemECs co-stained with Rhodamine conjugated phalloidin (red), DAPI (blue), and antibodies against phospho-FAK. Prop, propranolol.

Several reports have presented mixed results for the role of β-adrenergic receptor signaling in wound healing and cell migration, with evidence that inhibition of this class of receptors delays (16–19) or promotes (20–22) wound healing. Moreover, in cultured bovine aortic endothelial cells, β-adrenergic blockade with propranolol reportedly inhibits norepinephrine-induced induction of actin stress fibers (8), thus we sought to determine if similar effects on cell migration and cytoskeletal organization could be observed in HemECs treated with propranolol. HemECs, HDMVECs and HCAECs were grown to confluence, manually scratch wounded with a micropipette tip, and imaged using time lapse microscopy over a period of 12 h. As illustrated in Fig. 4A, treatment of HemECs with 50 μM propranolol resulted in a significant reduction in ‘wound’ closure compared to the control. Quantification of the time lapse images taken from the scratch migration assay revealed that propranolol dramatically reduced the migratory speed of HemECs (34% reduction), HMVECs (66% reduction) and HCAECs (67% reduction) (Fig. 4B), suggesting that propranolol is more effective at blocking proliferation of normal endothelial cells than HemECs. To identify signaling events that might shed light on how propranolol disrupts migration, we performed western blot analysis to detect the activation status of several known regulators of actin cytoskeletal dynamics including focal adhesion kinase (FAK), cofilin, ezrin/radixin/moesin (ERM), and myosin phosphatase (MYPT1), revealing that this drug effectively decreases the inhibitory phosphorylation of cofilin (an actin severing protein) at serine-3 (Fig. 4C). Given the increased activation of cofilin following propranolol treatment, we suspected that changes in the actin microfilament cytoskeleton would ensue. Indeed, immunofluorescent detection of actin stress fibers revealed that propranolol markedly inhibits actin polymerization in HemECs (Fig. 4D) and normal endothelial cells (data not shown) consistent with that expected from activation of cofilin. Computational analysis of actin stress fiber length using the FiberScore algorithm (Lichtenstein) demonstrated that propranolol-treated HemECs exhibit a greater than 1.5-fold reduction in average fiber length compared to the sham treatment (data not shown). Similar observations were observed for non-hemangioma endothelial cells (data not shown). In addition to altering actin stress fiber polymerization, propranolol shifted the subcellular localization of p-FAK from regions of colocalization with actin stress fibers in sham-treated cells to diffuse punctuate regions located throughout the cytoplasm in propranolol-treated cells (Fig. 4D). Despite alterations in p-FAK subcellular localization, no changes in the levels of p-FAK were observed in response to propranolol (Fig. 4C).

### Propranolol disrupts global gene expression patterns in endothelial cells

**Figure 5:**
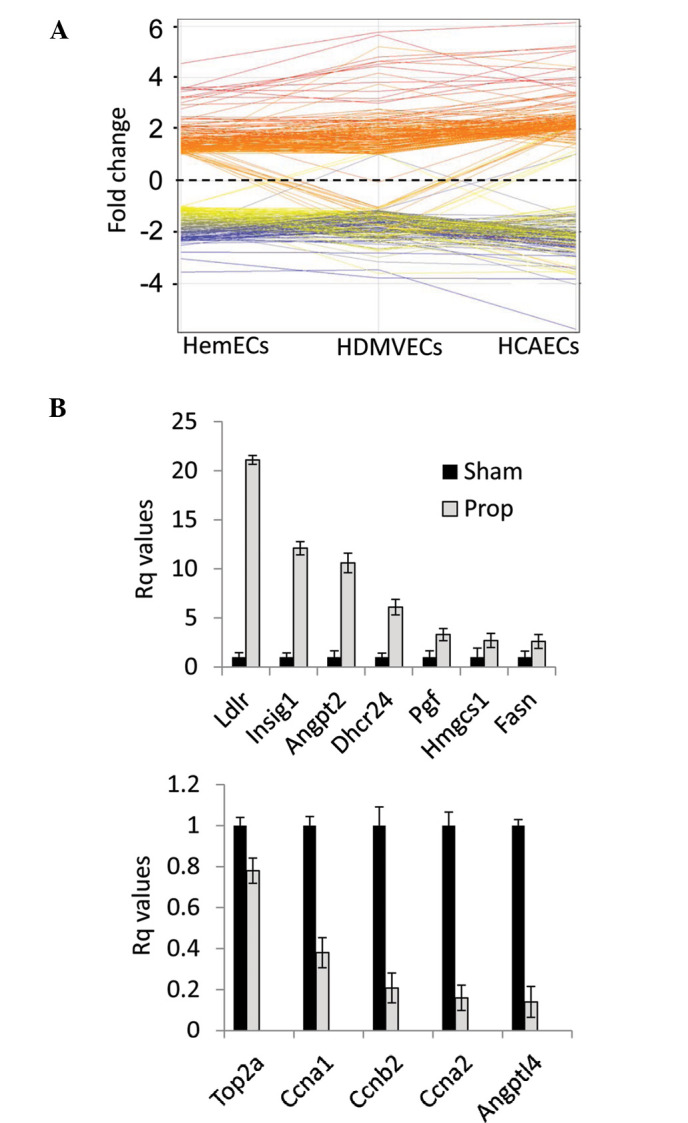
Propranolol induces significant alterations in global gene expression of human infantile hemangioma endothelial cells (HemECs). (A) Correlation map comparing the significant gene expression changes (>2 fold gene expression alteration, p<0.05) as determined by microarray analysis between HemECs, human dermal microvascular endothelial cells (HDMVECs), and human coronary artery endothelial cells (HCAECs) treated with sham or 50 μM propranolol for 24 h. (B) RT-PCR confirmation of a subset of genes in HemECs whose expression was statistically altered in the microarray.

Propranolol has been shown to affect the expression of cyclins across multiple cell types (5,23), gluconeogenic and glycolytic enzymes in the liver (24), epidermal growth factor 1 in cardiomyocytes (25) and pigment epithelial derived factor in the retina (26). However, these studies have focused on small subsets of genes and did not look at large-scale changes in genomic expression patterns. To evaluate the global gene expression changes in HemECs in response to propranolol and to examine how the identified changes compared with those observed in propranolol-treated normal human endothelial cells, we performed whole genome microarrays providing coverage for more than 47,000 transcripts and known splice variants across the human transcriptome. Our analysis identified 89 genes whose expression in HemECs was altered greater than 2-fold (p<0.05) in response to propranolol (32 genes significantly upregulated and 57 genes downregulated) (Table II). Several functional groupings of genes were identified included those involved in lipid and sterol metabolism (21% of total gene expression changes: *HMGCS1*, *MSMO1*, *LDLR*, *DHCR7*, *SCD*, *ACAT2*, *LSS*, *FASN*, *MSMO1*, *SQLE*, *DHCR24*, *FDFT1*, *IDI1*, *FADS2*, *ACSS2*, *EBP*, *SC5DL*, *NSDHL* and *LPIN1*), cell cycle regulation (14% of total gene expression changes: *CDC2*, *CDCA8*, *CDKN3*, *PRC1*, *MCM4*, *CDC20*, *CDC45L*, *CCNA1*, *CCNB2*, *CCNA2*, *TOP2A* and *CDCA7*), angiogenesis (5% of total gene expression changes: *PGF*, *ANGPT2*, *ANGPTL4* and *RGS4*) and ubiquitin modifications (3% of total gene expression changes: *UBE2T*, *UBE2C* and *UHRF1*). Comparative analysis of genes whose expression was statistically altered by propranolol treatment in any of the three endothelial cell line tests revealed a strong correlation between cell lines (Fig. 5A). This data indicate that propranolol-induced gene expression changes are endothelial cell-type independent. Quantitative RT-PCR validation of ∼14% of the propranolol-responsive genes in HemECs revealed comparable alterations in gene expression similar to that revealed by microarray analysis, thus corroborating our microarray data through independent analysis (Fig. 5B).

## Discussion

IHs as a whole are largely understudied considering the high prevalence of these lesions in children and the serious threat to health they pose in certain instances. To date, there remains a great deal of uncertainty as to the origin of these tumors, with evidence suggesting they may be caused by aberrant transplantation of placental endothelial cells (27), predisposing genetic factors (28,29) and/or tumor stem cell components (30). Despite the controversial origin of these tumors, proliferating IHs are characterized by an enhanced angiogenic capacity largely due to modulation of signaling pathways that regulate the VEGF signaling axis, while involuting IHs display a chronic inflammatory response and downregulation of angiogenesis regulators (31). The recent discovery that the β blocker propranolol is an effective treatment for IHs suggests that the sympathetic nervous system may play a key role in controlling IH growth. Epinephrine is a sympathomimetic amine that increases the activity of noradrenaline in post-synaptic cells, and is capable of enhancing vasodilation through activation of β-adrenergic receptors. β-adrenergic receptors have been shown to be expressed on normal capillary endothelial cells (32) and it is believed that β blockers, such as propranolol, inhibit epinephrine signaling through inducing vasoconstriction of endothelial cells and disruptions in key angiogenic processes (7–9,33). Moreover, β blockers have been shown to reduce the expression of VEGF in non-endothelial cells, thus leading to inhibition of angiogenic paracrine signaling (34). Despite the extensive use of propranolol in the treatment of IHs, very little has been done to determine the molecular mechanisms of propranolol in IH tumors. This drug is presumably believed to induce IH tumor regression through mechanisms similar to that noted in normal endothelial cells, and recent reports suggest that it may work in part through suppressing production of nitric oxide and HIF1α regulation of VEGF expression (10,11). In this study, we sought to investigate how propranolol disrupts HIHEC function and compare these effects to those seen in normal primary endothelial cell lines. Our data demonstrated that β-adrenergic receptors are expressed across a panel of HIHEC and normal endothelial cells. We further showed that propranolol disrupts HIHEC and normal endothelial cell cycle progression, migration, cytoskeletal dynamics, and gene expression, and we elucidated multiple downstream targets of propranolol including cell cycle progression regulators, cytoskeletal modulators and gene expression alterations.

The expression of β1- and β2-adrenergic receptors has been extensively studied in the cardiovascular system, with high expression occurring in cardiac myocytes and vascular smooth muscle cells (35). Although β3-knockout mice display increased hypotension in response to isoproternol (36), these receptors are suspected to play a lesser role in cardiovascular function compared to β1- and β2- receptors as they are expressed primarily in brown adipocytes, gallbladder and the colon (37). Our data indicate that both β1- and β2- (but not β3-) adrenergic receptors are expressed on primary cultures of HemECs. Interestingly, comparisons of the relative mRNA expression levels of these receptors on primary cultures of non-diseased endothelial cells revealed similar levels to that of HemECs, causing us to question if propranolol is selective for HemECs or if this drug demonstrates a similar level of inhibition for diseased and normal endothelial cells. Indeed, comparisons of the inhibitory effect of propranolol at its IC_50_ on cultures of HemECs, HDMVECs, and HCAECs revealed that this drug indiscriminately blocks proliferation, migration, and actin polymerization in an endothelial cell-type independent manner. This finding suggests that the mechanism of action for propranolol on IHs may extend beyond simply blocking endothelial cell function. For instance, IHs display a high pericyte density in the proliferating stage (38) and increased molecular markers of endothelial cell/pericyte interactions (31). Primary cultures of pericytes express functional adrenergic receptors and respond to autonomic vasoactive substances *in vivo* (39). As pericytes are responsible for a number of roles in the microvasculature including capillary maturation and stabilization, further studies should examine if propranolol inhibits IH growth through destabilization of endothelial cell/pericyte interactions. Another possibility that may account for the endothelial cell-type independent action of propranolol may have to do with the limitations of *in vitro* monolayer cell culture systems. Children undergoing systemic propranolol treatment for IHs often undergo side effects including bradycardia, hypotension and hypoglycemia (40), however significant disruptions of their existing vascular beds have not been reported. This suggests that propranolol may preferentially inhibit proliferating endothelial vasculature while sparing the quiescent established vasculature. However, this possibility is complicated by reports suggesting that propranolol improves wound healing - a process intimately dependent on neovascularization (20–22).

There is evidence that VEGFR-2 phosphorylation is controlled by β-adrenergic signaling (5,41,42), however the mechanisms underlying this effect remain to be determined. As HemECs are characterized by aberrant constitutive activation of VEGFR-2 signaling, we sought to determine whether propranolol is capable of attenuating this process. Similar to data reported in normal endothelial lines, propranolol effectively blocks VEGFR-2 phosphorylation on HemECs. We tested the effects of propranolol on known downstream targets of VEGF signaling revealing propranolol-induced alterations in p38 signaling, decreased cyclin expression and increased cyclin-dependent kinase inhibitor steady state mRNA levels. Moreover, propranolol treatment of the panel of endothelial cells resulted in reduced proliferation rates and increased percentages of cells in the G1 cell cycle phase. These alterations were coincident with significant changes in the levels of key cyclins and cell cycle inhibitors. Interestingly, of the panel of MAPK proteins that we tested, we saw increased phosphorylation only in p38, which is known to be responsive to stress stimuli such as cytokines, irradiation and shock. p38 plays a central role in inflammation and regulates the production of inflammatory mediators such as TNFα, IL1β, and COX2 (43), thus it is possible that increased p38 activation in propranolol-treated IHs may mediate IH regression through immune-mediated responses. A number of studies suggest that propranolol may induce apoptosis of HemECs. No caspase cleavage or apoptosis was observed at the IC_50_ (∼50 μM) for propranolol in HemECs or normal endothelial cells, although we did begin to see lethal effects of this treatment in all three endothelial cell lines at upwards of 150 μM. These findings do not rule out that propranolol-induced IH apoptosis plays a role in the efficacy of this treatment, but it does suggest that basic endothelial functions such as proliferation and migration display greater susceptibility to lower doses of propranolol than does apoptosis. We demonstrated that propranolol treatment of HemECs results in abolished stress fiber formation, and this effect may be due in part to decreased levels of phosphorylated cofilin following propranolol treatment. Cofilin is a cytoskeletal-binding protein critical for actin microfilament dynamics and reorganization by severing and depolymerizing actin filaments (44). Cofilin phosphorylation is an inhibitory event (45), thus the absence of stress fiber formation observed in propranolol-treated HemECs may be due in part to increased cofilin-mediated actin severing. This effect would certainly disrupt cell migration, and as the actin cytoskeleton is intimately tied to the regulation of cell cycle progression (46), may indirectly contribute to propranolol induced decreased cell proliferation.

As propranolol appears to work with great efficacy against IHs, similar inhibitory effects could potentially be observed in other vascular tumors such as angiosarcomas and Kaposi’s sarcomas. Indeed, propranolol has been tested in preclinical and clinical models of malignant tumors, demonstrating good efficacy in the treatment of melanoma (47), pancreatic (48), colorectal (49) and breast (50) cancer. Finally, given the cutaneous nature of IHs and the endothelial cell type-independent effects of propranolol observed in our study, topical delivery of β blockers (as opposed to systemic delivery) should be aggressively pursued. A controlled study of topical administration of the β blocker timolol on non-life threatening IHs revealed consistently good to moderate responses in 91.5% of infants (51), suggesting this therapy could specifically treat the lesion area while preventing potential collateral anti-vascular effects on the normal endothelium.

## Figures and Tables

**Table I:** Percentage of endothelial cells in each cell cycle phase.

Cells	Sham	Propranolol
HemECs		
G1	68±2.3	74±2.2
S	8±0.6	5±0.3
G2/M	24±2.7	21±1.1
HDMVECs		
G1	69±3.0	75±1.6
S	8±4.1	4±0
G2/M	22±3.9	20±4.5
HCAECs		
G1	71±1.4	76±1.7
S	5±1.6	3±1.3
G2/M	23±3.3	20±2.5

HemECs, human infantile hemangioma endothelial cells; HDMVECs, human dermal microvascular endothelial cells; HCAECs, human coronary artery endothelial cells.

**Table II:** Alterations in gene expression (fold-change) induced by propranolol treatment.

Gene symbol	Gene name	Accession no.	HIHEC	HDMVEC	HCAEC
*HMGCS1*	3-Hydroxy-3-methylglutaryl-CoA synthase 1	NM_002130.6	4.6	5.8	6.2
*MSMO1*	Methylsterol monooxygenase 1, TV2	NM_001017369.2	4.5	4.0	5.8
*INSIG1*	Insulin induced gene 1	NM_198336.2	4.3	4.6	4.9
*LDLR*	Low density lipoprotein receptor	NM_000527.4	3.6	3.8	3.9
*MVD*	Mevalonate decarboxylase	NM_002461.1	3.6	5.7	3.4
*DHCR7*	7-Dehydrocholesterol reductase	NM_001360.2	3.6	4.7	3.4
*SCD*	Stearoyl-CoA desaturase	NM_005063.4	3.3	3.2	5.0
*ACAT2*	Acetyl-CoA acetyltransferase 2	NM_005891.2	3.2	4.6	5.2
*LSS*	Lanosterol synthase	NM_002340.5	3.2	4.5	5.1
*TM7SF2*	Transmembrane 7 superfamily member 2	NM_003273.2	3.0	4.8	5.2
*HMGCR*	3-Hydroxy-3-methylglutaryl-CoA reductase	NM_000859.2	2.9	3.1	3.8
*FASN*	Fatty acid synthase	NM_004104.4	2.8	4.6	4.3
*MSMO1*	Methylsterol monooxygenase 1, TV1	NM_006745.4	2.8	2.0	3.0
*SQLE*	Squalene epoxidase	NM_003129.3	2.7	2.6	3.6
*PSG4*	Pregnancy specific β-1-glycoprotein 4	NM_002780.3	2.6	1.3	4.5
*DHCR24*	24-Dehydrocholesterol reductase	NM_014762.3	2.5	2.4	3.3
*FDFT1*	Farnesyl-diphosphate farnesyltransferase 1	NM_004462.3	2.5	2.2	3.2
*IDI1*	Isopentenyl-diphosphate δ isomerase 1	NM_004508.2	2.4	2.3	3.4
*FADS2*	Fatty acid desaturase 2	NM_004265.2	2.4	4.2	2.8
*NPC1*	Niemann-Pick disease, type C1	NM_000271.4	2.3	2.3	3.8
*PFKFB4*	Fructose-2,6-biphosphatase 4	NM_004567.2	2.2	2.8	2.3
*ACSS2*	Acyl-CoA synthetase family member 2, TV2	NM_001076552.2	2.2	2.6	3.5
*ACSS2*	Acyl-CoA synthetase family member 2, TV1	NM_018677.3	2.2	2.7	3.2
*EBP*	Emopamil binding protein	NM_006579.2	2.1	2.4	2.3
*LOC100129668*	LOC100129669	XM_001713607.1	2.1	2.2	2.5
*HMOX1*	Heme oxygenase (decycling) 1	NM_002133.2	2.1	2.1	3.5
*SC5DL*	Sterol-C5-desaturase-like	NM_006918.4	2.1	1.4	2.4
*NSDHL*	NAD(P) dependent steroid dehydrogenase-like	NM_015922.2	2.1	2.3	2.2
*P2RX4*	Purinergic receptor P2X, 4	NM_002560.2	2.1	1.7	1.8
*LPIN1*	Lipin 1	NM_145693.1	2.0	1.6	2.9
*PGF*	Placental growth factor	NM_002632.5	2.0	1.7	2.3
*ANGPT2*	Angiopoietin 2	NM_001147.2	2.0	1.0	2.1
*LOC729009*	LOC729010	XR_042330.1	2.0	1.6	2.6
*IL8*	Interleukin 8	NM_000584.3	−2.0	−2.9	−3.1
*CDK1*	Cyclin-dependent kinase 1	NM_001786.4	−2.0	−1.8	−2.1
*TUBB2C*	Tubulin, β 4B Ivb	NM_006088.5	−2.0	−1.4	−2.5
*PTTG1*	Pituitary tumor-transforming 1	NM_004219.2	−2.0	−2.0	−2.0
*OIP5*	Opa interacting protein 5	NM_007280.1	−2.0	−2.3	−2.1
*CDCA8*	Cell division cycle associated 8	NM_018101.3	2.0	−1.4	−2.3
*TAGLN*	Transgelin	NM_003186.3	−2.0	−1.7	−2.9
*CDKN3*	Cyclin-dependent kinase inhibitor 3	NM_005192.3	−2.0	−1.7	−1.6
*ANLN*	Anillin, actin binding protein	NM_018685.2	−2.0	−1.8	−1.7
*HJURP*	Holliday junction recognition protein	NM_018410.3	−2.0	−1.2	−2.0
*PBK*	PDZ binding kinase	NM_018492.2	−2.0	−1.6	−2.5
*UBE2T*	Ubiquitin-conjugating enzyme E2T (putative)	NM_014176.3	−2.0	−1.4	−1.5
*STEAP1*	6-Transmembrane epithelial antigen of the prostate 1	NM_012449.2	−2.0	−1.8	−1.8
UBE2C	Ubiquitin-conjugating enzyme E2C, TV3	NM_181800.1	−2.0	−1.9	−2.8
CKS1B	CDC28 protein kinase regulatory subunit 1B	NM_001826.2	−2.0	−1.6	−1.9
TACC3	Transforming, acidic coiled-coil containing protein 3	NM_006342.2	−2.0	−1.4	−1.8
NCAPG	Non-SMC condensin I complex, subunit G	NM_022346.3	−2.0	−1.7	−1.6
PCDH7	Protocadherin 7	NM_002589.2	−2.0	−1.2	−2.2
FAM64A	Family with sequence similarity 64, member A	NM_019013.2	−2.1	−1.2	−2.0
PRC1	Protein regulator of cytokinesis 1	NM_199413.1	−2.1	−1.5	−2.3
MELK	Maternal embryonic leucine zipper kinase	NM_014791.3	−2.1	−1.8	−2.1
TPX2	TPX2, microtubule-associated	NM_012112.4	−2.1	−1.4	−2.2
MCM4	Minichromosome maintenance complex CMPT 4, TV2	NM_182746.2	−2.1	−1.6	−1.9
*ZWINT*	ZW10 interactor	NM_001005413.1	−2.1	−1.5	−2.0
*KIFC1*	Kinesin family member C1	NM_002263.2	−2.1	−1.4	−2.2
*CDC20*	Cell division cycle 20	NM_001255.2	−2.2	−2.0	−4.0
*UBE2C*	Ubiquitin-conjugating enzyme E2C, TV6	NM_181803.1	−2.2	−1.8	−2.4
*NCAPG2*	Non-SMC condensin II complex, subunit G2	NM_017760.5	−2.2	−1.3	−1.4
*SERPIND1*	Serpin peptidase inhibitor, clade D, member 1	NM_000185.3	−2.2	−1.8	−2.6
*CDC45L*	Cell division cycle 45	NM_003504.3	−2.2	−1.8	−3.0
*LYAR*	Ly1 antibody reactive	NM_017816.2	−2.2	−1.6	−1.3
*CCNA1*	Cyclin A1	NM_003914.3	−2.2	−2.2	−1.9
*TRIP1*	Thyroid hormone receptor interactor 13	NM_004237.3	−2.2	−1.9	−2.6
*MPZL2*	Myelin protein zero-like 2, TV2	NM_144765.2	−2.2	−1.3	−2.4
*CEP55*	Centrosomal protein 55kDa	NM_018131.4	−2.2	−2.0	−2.1
*CXCL1*	Chemokine (C-X-C motif) ligand 1	NM_001511.3	−2.2	−2.5	−2.8
*CCNB2*	Cyclin B2	NM_004701.3	−2.2	−2.2	−2.3
*KIF20A*	Kinesin family member 20A	NM_005733.2	−2.2	−1.9	−2.5
*RAD51AP1*	RAD51 associated protein 1	NM_006479.4	−2.2	−1.6	−2.2
*GINS2*	GINS complex subunit 2	NM_016095.2	−2.2	−1.7	−2.9
*FAM83D*	Family with sequence similarity 83, member D	NM_030919.2	−2.3	−1.6	−2.4
*KIAA0101*	KIAA0101	NM_014736.4	−2.3	−2.0	−2.3
*DLGAP5*	Discs, large (Drosophila) homolog-associated protein 5	NM_014750.4	−2.3	−2.0	−2.3
*CCNA2*	Cyclin A2	NM_001237.3	−2.3	−2.2	−2.8
*LOC399942*	LOC399943	XM_934471.1	−2.3	−2.4	−3.4
*MPZL2*	Myelin protein zero-like 2, TV1	NM_005797.3	−2.3	−2.7	−2.2
*TOP2A*	Topoisomerase II α 170kDa	NM_001067.3	−2.3	−2.4	−2.6
*RRM2*	Ribonucleotide reductase M2	NM_001034.3	−2.3	−1.5	−2.8
*FBXO5*	F-box protein 5	NM_012177.3	−2.4	−1.9	−2.6
*CDCA7*	Cell division cycle associated 7	NM_031942.4	−2.4	−1.9	−2.5
*MCM4*	Minichromosome maintenance complex CMPT 4, TV1	NM_005914.3	−2.4	−1.5	−2.0
*MAD2L1*	MAD2 mitotic arrest deficient-like 1	NM_002358.3	−2.4	−2.3	−2.1
*UHRF1*	Ubiquitin-like with PHD and ring finger domains 1	NM_001048201.1	−2.5	−1.6	−2.6
*ANGPTL4*	Angiopoietin-like 4	NM_139314.1	−2.8	−2.8	−2.9
*RGS4*	Regulator of G-protein signaling 4	NM_005613.5	−3.0	−3.8	−3.8
*IL1RL1*	Interleukin 1 receptor-like 1	NM_003856.2	−3.2	3.4	−5.2

HDMVEC, human dermal microvascular endothelial cells; HCAEC, human coronary artery endothelial cells.
